# Land Use/Land Cover Classification and Its Variability Along the Jiangsu Coast Based on Pearson-SHAP-RFE Feature Selection from Landsat Imagery

**DOI:** 10.3390/s26185915

**Published:** 2026-09-18

**Authors:** Shuyuan Wang, Wentai Pang, Shuanggen Jin

**Affiliations:** 1 School of Remote Sensing and Geomatics Engineering, Nanjing University of Information Science and Technology, Nanjing 210044, China; 202312480161@nuist.edu.cn; 2 Inner Mongolia Academy of Science and Technology, Hohhot 010010, China; imast_rlb@imast.ac.cn; 3 School of Geomatics and Aerospace Information, Henan Polytechnic University, Jiaozuo 454003, China; 4 School of Artificial Intelligence, Anhui University, Hefei 230601, China

**Keywords:** LULC, coastal zone, Landsat, Pearson-SHAP-RFE, feature selection

## Abstract

The Jiangsu coastal zone has experienced substantial land use/land cover (LULC) changes under intensified anthropogenic activities, while spectral confusion caused by land–sea interactions increases the difficulty of accurate LULC classification. This study investigated LULC dynamics in the Jiangsu coastal zone in 2000, 2008, 2016, and 2024 using Landsat imagery and the Google Earth Engine platform. A total of 33 features, including spectral bands, spectral indices, texture, topographic variables, and nighttime light data, were extracted. To improve classification accuracy and model generalization, a Pearson-SHAP-RFE feature selection framework was developed by integrating Pearson correlation analysis, SHapley Additive exPlanations, and recursive feature elimination. Four classification algorithms, namely Random Forest, Extreme Gradient Boosting, Classification and Regression Trees, and Support Vector Machine, were compared. Feature selection improved classification accuracy across the four algorithms, with improvements ranging from 4.05% to 5.15% compared with the spectral feature baseline. Random Forest achieved the highest overall accuracy of 92.28%, with a Kappa coefficient of 0.9019. The long-term classification results showed that LULC changes mainly occurred among forest–grassland, construction land, and cultivated land. Water bodies showed the smallest centroid shift, whereas cultivated land exhibited the greatest spatial displacement.

## 1. Introduction

The coastal zone of Jiangsu Province represents a critical ecotone characterized by a unique geographical location and abundant natural resources, establishing it as a vital industrial and agricultural base in eastern China [1]. Over the past two decades, regional strategies including “Maritime Jiangsu East” and “Coastal Development initiatives” have catalyzed rapid economic expansion in these coastal areas [2], which collectively accelerate industrialization and urbanization [3]. Concomitantly, this growth has precipitated a surge in the demand for water and land resources, triggering profound change in land use patterns [4]. These intensive development pressures have, in turn, driven the loss of tidal flat wetlands, the degradation of ecosystems, the overexploitation of land resources, and the intensified environmental pollution. Therefore, accurate land cover classification is essential for understanding land use dynamics and supporting sustainable coastal development.

The proliferation of remote sensing technology and image processing techniques has established satellite imagery as a primary tool for land use/land cover (LULC) studies [5]. Nevertheless, traditional remote sensing image processing, often dependent on local computing resources, is frequently hampered by challenges such as high data storage requirements, complex processing workflows, and computational inefficiency. In contrast, Google Earth Engine (GEE) provides cloud computing capabilities and extensive remote sensing data resources, substantially simplifying data preprocessing and analysis [6]. Parallel to these platform advancements, classification research has progressively shifted from relying on single features to integrating multi-source features, a methodological evolution that is critical for enhancing classification accuracy and model robustness. Early studies mainly relied on spectral information derived from satellite imagery. However, persistent challenges like intra-class spectral variability and inter-class spectral similarity have limited the accuracy of classification based solely on spectral data [7]. In response, a growing body of research has integrated ancillary data, such as terrain and texture features, to improve classification performance. Liang et al. [8]. demonstrated that integrating texture, topographic, and nighttime light features into the summer and winter land cover classification model for the Xinjiang region yielded an overall classification accuracy of 95.6% in summer and 91.3% in winter. Hänsch and Hellwich [9] utilized RF classifier for urban land cover classification, attaining an overall accuracy of 65.90% through the integration of Digital Surface Models (DSM), LiDAR intensity characteristics, and hyperspectral data. Wu et al. [10] developed an object-oriented composite classification model based on remote sensing imagery from the ZY1-02D satellite’s VNIC and AHSI cameras, extracting multi-source feature information including spectral, edge shape, and texture features. The model significantly improved classification accuracy, with a kappa coefficient reaching 0.846.

However, the incorporation of multi-source features, although significantly enriching the information content, inevitably introduces challenges including feature redundancy and the Hughes effect [11]. Such challenges can result in model overfitting and decreased computational efficiency, thereby impairing the stability and generalization capability of the models. Consequently, data dimensionality reduction—comprising feature extraction and feature selection—is essential. Although feature extraction methods such as PCA can reduce redundancy, the resulting components often lack clear physical meaning. Conversely, feature selection retains the most representative original feature subsets, preserving physical interpretability and explicitly identifying feature contributions. Due to its superior applicability, it has increasingly become the dominant approach for dimensionality reduction [12]. Zhao et al. [13] employed the RF out-of-bag (OOB) error for feature selection, recognizing optimal feature combinations to perform fine-grained grassland classification. Their methodology yielded significantly improved computational efficiency alongside a 3% increase in classification accuracy. Chao et al. [14] proposed a remote sensing feature parameter optimization method based on the Gini index of random forests, using a 10% threshold criterion to select the optimal parameter combination and achieve the best land cover classification results. A key limitation of such global importance metrics, however, is their tendency to evaluate only a feature’s overall contribution to model performance, overlooking its category-specific predictive influence [15]. Meanwhile, in coastal environments characterized by highly fragmented landscapes and substantial spectral overlap among LULC types, conventional dimensionality reduction approaches may have difficulty identifying the key variables that distinguish specific classes, potentially resulting in the loss of physically meaningful information and limiting further improvements in refined classification and physical interpretation. SHapley Additive exPlanations (SHAP), a widely recognized post-hoc machine learning interpretability framework [16,17,18], addresses this gap by not only quantifying overall feature importance but also disaggregating each feature’s contribution to model predictions. Despite its interpretive power, existing research has mainly focused on applying SHAP to explain decision-making processes, while its potential for driving feature selection to optimize LULC classification models remains relatively underexplored.

To address these research limitations, this study focuses on how to effectively select features from high-dimensional multi-source data in coastal environments and further reveal the discriminative roles of key features for different LULC classes, thereby developing a LULC classification framework that integrates classification performance with interpretability. Accordingly, the coastal zone of Jiangsu Province was selected as the study area. Using the GEE platform, multi-source remote sensing imagery was obtained to systematically construct multi-dimensional feature sets, including spectral data, spectral indices, texture, topography, and nighttime light information. We hereby propose a novel hybrid feature selection framework that synergistically combines the Pearson correlation coefficient, SHAP importance scores, and recursive feature elimination (RFE). Unlike global importance metrics that only provide a generalized ranking, this framework leverages the additive explanation property of SHAP to identify both the most critical features for overall accuracy and the category-specific predictive influence of variables, thereby enhancing the discrimination of spectrally similar LULC classes in complex coastal ecotones. A total of seven feature combination schemes were designed and coupled with four classical classification algorithms to perform LULC classification. Through a comparative analysis of the classification accuracies achieved by different feature combinations and algorithms, the optimal feature set and classification model were determined. Ultimately, this optimized classification approach was applied to track and quantify long-term LULC changes in the coastal zone of Jiangsu Province, furnishing precise and reliable data to inform coastal LULC monitoring and sustainable development.

## 2. Study Area and Data

### 2.1. Study Area

**Figure 1:**
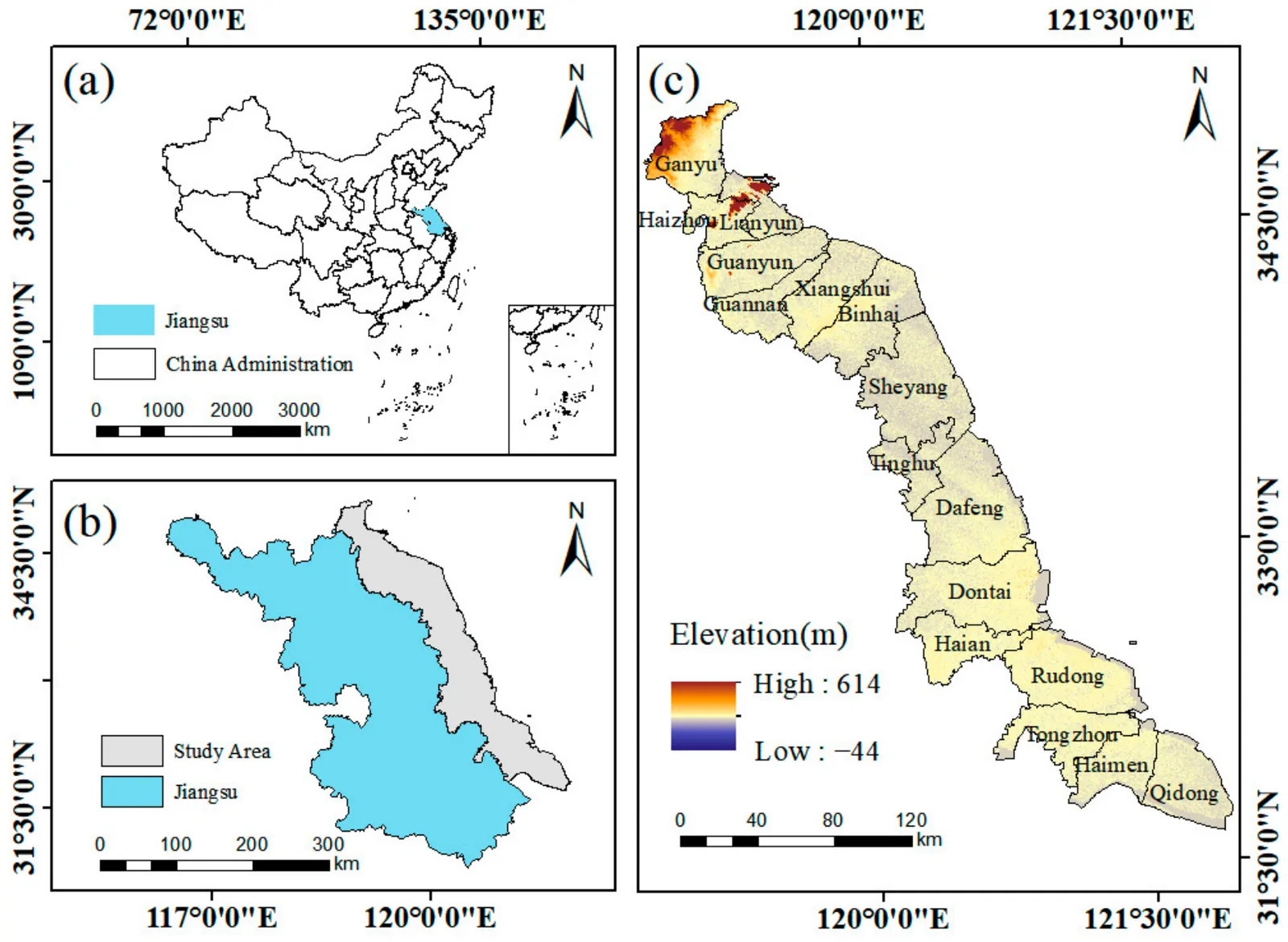
The study area of the research: (**a**) Location of Jiangsu Province; (**b**) Location of Jiangsu coastal zone; (**c**) Administrative divisions of the study domain with surface altitude.

The study domain including the entire coastal zone of Jiangsu Province was selected to conduct an intensive investigation of LULC classification, encompassing the coastal counties (cities, districts) under the jurisdiction of three prefecture-level cities, namely Lianyungang, Yancheng and Nantong (Figure 1). Geographically, this region spans 32°04′–35°12′ N and 119°15′–122°34′ E, positioning it as a strategic nexus between the national “Belt and Road” Initiative and the Yangtze River Economic Belt development strategy. The study area is predominantly flat, with elevations ranging from −44 to 614 m and higher terrain in the north. Climatically, the region experiences a subtropical and warm temperate monsoon climate, which provides abundant sunlight and an annual average temperature ranging from 13.5 to 16.0 °C. The predominant land type is cultivated land, accounting for about 70% of the total area, forming strong landscape matrix of the region [19]. According to the Jiangsu Statistical Yearbooks, over the past decade, the regional gross domestic product (GDP) increased from 866.283 billion RMB to 1981.424 billion RMB. By 2024, the region’s population was 19.0027 million. Driven by the concerted advancement of the national coastal development and ecological civilization construction strategies, the Jiangsu coastal zone has emerged as one of the most economically dynamic and intensively developed regions within Jiangsu Province. However, human activities and rapid economic development have posed challenges to the ecological civilization of the Jiangsu coastal zone, leading to the deterioration of the ecological environment [20]. Therefore, to carry out land classification in the Jiangsu coastal zone, accurate basic LULC data are urgently needed to provide support for ecological protection, planning and regulation, and sustainable development.

### 2.2. Data Sources

(1) Landsat Data: The remote sensing imagery used in this study was derived from the Landsat satellite series. The Landsat satellite series provides long-term, globally available Earth observation data at medium spatial resolution [21]. The remote sensing imagery used in this study consisted of Landsat surface reflectance products for 2000, 2008, 2016, and 2024. Landsat 5 was used for 2000 and 2008, whereas Landsat 8 was used for 2016 and 2024, and all data were provided by USGS and accessed through the GEE platform (https://earthengine.google.com; accessed on 15 October 2025). This data has undergone official preprocessing procedures such as atmospheric correction and radiometric correction in advance, ensuring high data quality [22].

(2) Terrain Data: The Shuttle Radar Topography Mission (SRTM) data is a product jointly developed by the National Aeronautics and Space Administration (NASA) and international partners [23], aiming to create a global Digital Elevation Model (DEM) through radar technology. The SRTM V3 product provided by the GEE platform was used as the terrain feature input for training the model in this study, with a spatial resolution of 30 m.

(3) Nighttime Light Data: To improve classification accuracy in the coastal zone, the study also used two nighttime light products based on the GEE platform: DMSP-OLS Nighttime Lights Time Series during the period 2000–2008 from the Defense Meteorological Satellite Program (DMSP), and NPP-VIIRS Day/Night Band (DNB) during the period 2012–2024 from the Suomi National Polar-Orbiting Partnership (Suomi NPP), with spatial resolutions of 900 and 450 m, respectively [24].

### 2.3. Data Preprocessing

Leveraging the GEE platform, Landsat surface reflectance images were preprocessed for four target years, namely 2000, 2008, 2016, and 2024. For each target year, images with scene-level cloud cover below 20% were first selected and clipped to the study area boundary. All available images meeting the image quality requirements throughout the year were then composited using the median method to generate representative annual images and reduce the influence of clouds and anomalous observations. Finally, the processed annual composite images were exported for subsequent analysis.

The “ee.Algorithms.Terrain” function in the GEE platform was used to calculate the SRTM DEM data to obtain the terrain features of the study area, namely elevation, slope, aspect and hillshade. Meanwhile, the coarse-resolution DMSP-OLS/NPP-VIIRS nighttime light data were resampled to a 30 m resolution using bilinear interpolation and aligned to the same grid.

## 3. Methodology

**Figure 2:**
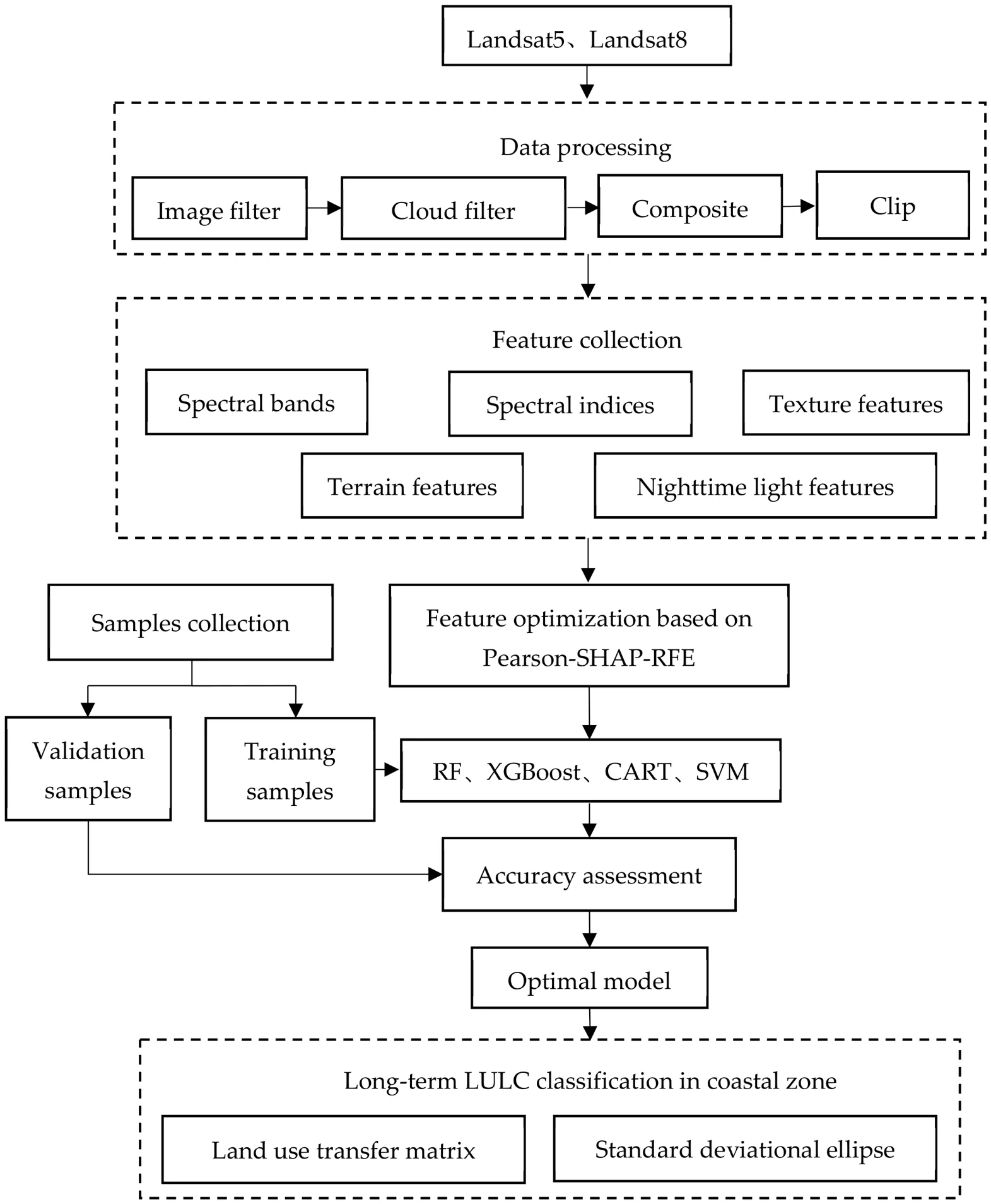
Workflow of this study.

The overall technical workflow of this study is illustrated in Figure 2. GEE was used for Landsat image preprocessing, multi-source feature construction, and sample selection and labeling, whereas subsequent feature selection, model training, and accuracy assessment were performed in a local Python 3.10 environment. Within this workflow, a novel hybrid feature selection framework termed Pearson–SHAP–RFE was proposed. The methodology then proceeded with an initial feature screening by integrating the Pearson correlation coefficient with SHAP importance to discriminate features characterized by high redundancy and low predictive importance. Building upon this, a recursive feature elimination algorithm was introduced for iterative refinement, ultimately yielding the optimal feature subset. To rigorously evaluate the efficacy of the feature selection process and the adaptability of the models, seven distinct feature combination schemes were devised and tested using four classification algorithms, namely RF, XGBoost, CART, and SVM. Through multiple groups of comparative experiments, the classification performance of each feature combination under different classifiers was systematically analyzed, and the optimal classification model was then determined.

### 3.1. Feature Set Construction

This study integrated multi-source data to extract five types of features, comprising a total of 33 variables, including original spectral bands, spectral indices and texture features of multispectral images, nighttime light features, and topographic features derived from SRTM data. Specifically, 11 spectral indices were calculated on the GEE platform, and their calculation formulas are provided in Table 1. For texture feature extraction, the commonly used Gray-Level Co-occurrence Matrix (GLCM) was selected. The GLCM effectively characterizes the spatial correlation between pixels and captures the statistical properties of image tone. We extracted ten widely used parameters including Mean (MEAN), Variance (VAR), Contrast (CON), Dissimilarity (DIS), Entropy (ENT), Angular Second Moment (ASM), Correlation (COR), Inverse Difference Moment (IDM), Cluster Shade (SHADE), and Cluster Prominence (PROM).

### 3.2. Classification Algorithms

The RF algorithm is an ensemble machine learning method that constructs multiple, mutually independent classification decision trees for classification [36]. Using the Bagging method, RF generates a large number of decision trees, with the final classification outcome determined by majority voting [37]. A key advantage of RF is its ability to mitigate the overfitting problem inherent in single decision trees. The algorithm also demonstrates good tolerance to noise and outliers, coupled with excellent scalability and parallelism for high-dimensional data classification. Referring to the parameter settings used in previous remote sensing classification studies [38,39], the number of trees (n_estimators) was evaluated from 30 to 200 with a step size of 5. The highest classification accuracy was obtained with 185 trees, which was therefore adopted for the final RF model. The number of features considered at each split (mtry) was set to its default value, corresponding to the square root of the total number of input features.

The CART algorithm is a classic decision tree system, proposed by American scientist Breiman [40]. It is well-suited for spatial mining and knowledge discovery, offering a clear structure, computational efficiency, and broad application in the research of remote sensing image information extraction and classification. The algorithm operates recursively by splitting nodes until it reaches the terminal nodes, based on a predefined threshold. In this approach, input data are split into group sets and the trees are constructed utilizing all except one of those. The tree is validated using the left-out group, and the reduced tree with the lowest deviation is selected [41]. CART performs effectively in handling complex datasets containing categorical and continuous variables, rendering it suitable for LULC classification [42]. The maximum depth of the tree is a key tuning parameter in CART that controls model complexity [43]. In this study, max_depth was evaluated from 5 to 9, and the value yielding the highest classification accuracy (max_depth = 7) was adopted for the final model.

The XGBoost is another powerful ensemble algorithm based on decision trees. It leverages CART as its base classifier, progressively adding trees to refine the model [44]. XGBoost constructs decision trees sequentially, with each subsequent tree correcting the errors of the previous ones by focusing on misclassified samples [45]. The algorithm minimizes a loss function through gradient descent, adjusting the weights of the trees iteratively to improve accuracy. A significant feature of XGBoost is its automated use of CPU multi-threading for parallel processing, leading to substantial gains in computational efficiency and accuracy. The algorithm remains highly efficient even with large-scale, high-dimensional remote sensing data analysis [46]. For XGBoost, n_estimators was evaluated from 200 to 500 in increments of 50 using five-fold cross validation on the training set, and 400 trees were selected. The final model used a learning rate of 0.04 and a maximum tree depth of 3.

Support Vector Machine (SVM) is a supervised non-parametric learning algorithm. Its basic principle is to find a hyperplane in the feature space that separates different classes while maximizing the margin between the hyperplane and the nearest training samples. When the input data are not linearly separable, kernel functions can be used to map the original data into a higher-dimensional feature space, thereby improving class separability. Owing to its ability to effectively handle high-dimensional feature spaces and achieve good generalization performance with relatively limited training samples, SVM has been widely applied in remote sensing classification tasks [47,48]. The following settings were used for the SVM classification: the kernel type was the radial basis function (RBF), and the kernel coefficient gamma and regularization parameter C were optimized using grid search with five-fold cross validation. The optimal values were determined as C = 10 and gamma = 0.01.

### 3.3. Feature Selection

In machine learning, the use of a full feature set often induces the “curse of dimensionality” [49]. Feature redundancy can increase model complexity, amplify sensitivity to noise, and ultimately lead to overfitting and performance degradation. The 2023 dataset was used for feature selection, model training, and performance evaluation. A Pearson–SHAP–RFE feature selection procedure was then implemented. First, Pearson correlation coefficients were calculated among all candidate features to identify highly correlated feature pairs. Second, the mean absolute SHAP value of each feature was calculated to quantify its contribution to model prediction. For each feature pair with an absolute Pearson correlation coefficient greater than 0.8, the feature with the lower mean absolute SHAP value was removed, thereby reducing feature redundancy while retaining the feature with greater predictive contribution. Finally, the remaining features were further screened using RFE, which iteratively removed the lowest-ranked feature and evaluated the classification performance of the resulting feature subsets to determine the optimal feature combination for LULC classification.

#### 3.3.1. Pearson Correlation Coefficient

The Pearson correlation coefficient quantifies the linear relationship between two continuous variables. The strength of the correlation is indicated by the absolute value of the coefficient, which ranges from −1 to 1. The calculation formula for the correlation coefficient *r* between variables *X* and *Y* is as follows:

(1) $$r=\frac{\sum_{i=1}^{n}\left(X_{i}-\bar{X}\right)(Y_{i}-\bar{Y})}{\sqrt{\sum_{i=1}^{n}{\left(X_{i}-\bar{X}\right)}^{2}}\sqrt{\sum_{i=1}^{n}{\left(Y_{i}-\bar{Y}\right)}^{2}}}$$

In the formula, *r* represents the correlation coefficient, *n* denotes the number of samples, $X_{i}$ and ${\;Y}_{i}$ are the observed values of variables *X* and *Y* for the *i*-th sample, respectively, while $\bar{X}$ and $\bar{Y}$ denote the sample means of variables *X* and *Y*, respectively. Previous remote sensing classification studies have commonly adopted Pearson correlation thresholds ranging from 0.7 to 0.9 to identify and remove highly correlated features and thereby reduce feature redundancy [50,51,52]. Accordingly, ∣*r*∣ = 0.7, 0.8, and 0.9 were selected for sensitivity analysis in this study, and five-fold cross validation was used to evaluate the classification performance of the feature subsets obtained after the preliminary Pearson–SHAP screening. The mean OAs were 0.8894, 0.9163, and 0.9226 for thresholds of 0.7, 0.8, and 0.9, respectively. After subsequent RFE, 8, 15, and 21 features were retained, respectively. Increasing the threshold from 0.7 to 0.8 improved the mean OA by 2.69 percentage points, whereas a further increase from 0.8 to 0.9 yielded only a 0.63 percentage-point improvement while increasing the final number of retained features from 15 to 21. Therefore, considering both classification performance and model simplification, ∣*r*∣ = 0.8 was adopted as the Pearson correlation threshold in this study. 

#### 3.3.2. SHAP Interpretable Framework

SHAP is a unified framework for interpreting the output of machine learning models, rooted in cooperative game theory’s Shapley value concept proposed by Lloyd Shapley in 1954 [53]. This application provides both local and global model interpretability. The SHAP value assigned to an individual feature reflects its marginal contribution to a specific model’s prediction results. For global feature importance ranking, which is particularly relevant for nonlinear models, the mean absolute SHAP value is calculated for each feature across all samples. A higher mean absolute value indicates a greater overall importance of the feature in the model’s decision-making. The relevant formula is as follows:

(2) $$g\left(z^{\prime }\right)=\varphi_{0}+\sum_{i=1}^{M}\varphi_{i}z_{i}^{\prime }$$

In the formula, $z^{\prime }\in {\left\{0,1\right\}}^{M}$ represents the presence or absence of a feature variable, where *M* denotes the number of feature variables in the model. $\varphi_{0}$ represents the constant when all inputs are missing. $\varphi_{i}$ represents the marginal contribution of variable *i*, i.e., the Shapley value.

#### 3.3.3. Recursive Feature Elimination (RFE)

The RFE is a wrapper-type feature selection algorithm that operates as a greedy, backward-elimination process. The goal of RFE is to iteratively remove features with the lowest predictive contributions to classification, thereby reducing redundancy and enhancing classifier performance. At each iteration, the model is trained using the current feature set, the features are ranked according to their importance, and the feature with the lowest importance is removed. The model is then retrained using the reduced feature set, and this process is repeated until the optimal feature subset is determined according to classification performance [54].

### 3.4. Different Dimensionality Reduction Methods

In order to demonstrate the effectiveness of the Pearson-SHAP-RFE framework in mitigating high-dimensional redundancy and the Hughes effect, comparative experiments were conducted using benchmark methods selected from the two dominant dimensionality reduction paradigms: feature selection and feature extraction.

(1) Pearson-Gini-RFE: The method utilizes the Mean Decrease in Gini (MDG) to measure feature importance. In detail, MDG quantifies the average decrease in Gini Impurity brought by a feature across all trees in the Random Forest, serving as an indicator of its predictive power. Operating under an RFE iterative framework consistent with the proposed method, it achieves optimal feature subset selection by successively removing features with lower contribution scores [55].

(2) Principal Component Analysis (PCA): The PCA is employed to reduce data dimensionality by condensing redundant information into a smaller set of orthogonal bands. These generated components are uncorrelated and ranked in descending order based on their variance contribution [56]. In the experiment, by setting a cumulative variance explained threshold of 95%, 12 principal components were ultimately extracted as input features for the model.

### 3.5. Accuracy Assessment

A rigorous accuracy assessment is essential for validating the reliability of LULC classification results. This study employed a confusion matrix to validate the accuracy of the LULC classification results. The confusion matrix is an N × N contingency table that compares reference data with classification outcomes. To ensure a comprehensive evaluation, several metrics such as Overall Accuracy (OA), Producer’s Accuracy (PA), User’s Accuracy (UA), F1-score, and Kappa Coefficient (KC) were selected. These metrics facilitate an objective and nuanced analysis of model performance, providing critical insights for model optimization and improvement. To quantify the uncertainty in the classification performance estimates, non-parametric bootstrap resampling of the test set with 1000 iterations was used to derive 95% confidence intervals for OA, Kappa, and F1-score. For the subsequent assessment of statistical significance between models, OA was designated as the primary comparison metric. Since all classifiers were evaluated using the same test samples, paired bootstrap resampling was further used to estimate the pairwise differences in OA (ΔOA) and their corresponding 95% confidence intervals. For each pairwise comparison, a two-sided bootstrap *p*-value was also calculated as twice the smaller tail proportion of replicates on either side of zero. A difference was considered statistically significant when its corresponding 95% confidence interval did not include zero [57].

### 3.6. Spatiotemporal Changes

This study reveals the spatiotemporal characteristics in land use change in the coastal area of Jiangsu Province using the land use transfer matrix, Sankey diagram, and standard deviational ellipse. The land use transfer matrix quantifies the conversion relationship between different LULC classes, thereby elucidating the structural characteristics of land use change over time. Its formula is as follows:

(3) $$L_{ab}=\left[\begin{array}{cccc} L_{11} & L_{12} & \cdots & L_{1n}\\ L_{21} & L_{22} & \cdots & L_{2n}\\ \vdots & \vdots & \vdots & \vdots \\ L_{n1} & L_{n2} & \cdots & L_{nn} \end{array}\right]$$

In the formula, $L_{ab}$ denotes the transfer area, where *a* represents the initial LULC class, *b* represents the final LULC class, *L* denotes the area size, and *n* denotes the number of LULC classes before and after conversion.

The Standard Deviational Ellipse (SDE) is a spatial statistical tool for analyzing the distributional characteristics of geographic features. This method provides a multi-dimensional summary of a feature’s spatial distribution based on the location and structure. It quantifies these characteristics using several core parameters, such as the centroid, azimuth, major axis, and minor axis. Collectively, these parameters (azimuth θ, the mean center, and the standard deviations) define the SDE and enable an effective analysis of the central tendency, dispersion, and orientation of geographical elements.

Expression for the azimuth:

(4) $$\tan\theta =\frac{\left(\sum_{i=1}^{n}w_{i}^{2}{\tilde{x_{i}}}^{2}-\sum_{i=1}^{n}w_{i}^{2}{\tilde{y_{i}}}^{2}\right)+\sqrt{{\left(\sum_{i=1}^{n}w_{i}^{2}{\tilde{x_{i}}}^{2}-\sum_{i=1}^{n}w_{i}^{2}{\tilde{y_{i}}}^{2}\right)}^{2}+4\sum_{i=1}^{n}w_{i}^{2}{\tilde{x_{i}}}^{2}{\tilde{y_{i}}}^{2}}}{2\sum_{i=1}^{n}w_{i}^{2}\tilde{x_{i}}\tilde{y_{i}}}$$

Average center formula:

(5) $$\bar{X_{w}}=\frac{\sum_{i=1}^{n}w_{i}x_{i}}{\sum_{i=1}^{n}w_{i}};\bar{Y_{w}}=\frac{\sum_{i=1}^{n}w_{i}y_{i}}{\sum_{i=1}^{n}w_{i}}$$

Standard deviations of the *x* and *y* axes:

(6) $$\sigma_{x}=\sqrt{\frac{\sum_{i=1}^{n}{\left(w_{i}\tilde{x_{i}}\cos\theta -w_{i}\tilde{y_{i}}\sin\theta \right)}^{2}}{\sum_{i=1}^{n}w_{i}^{2}}}$$

(7) $$\sigma_{y}=\sqrt{\frac{\sum_{i=1}^{n}{\left(w_{i}\tilde{x_{i}}\sin\theta -w_{i}\tilde{y_{i}}\cos\theta \right)}^{2}}{\sum_{i=1}^{n}w_{i}^{2}}}$$

In the formula:

*θ*—Azimuth angle;

$w_{i}$—Weight of point *i*;

$\left(x_{i},y_{i}\right)$—Spatial location of the research object;

$\left(\bar{X_{w}},\bar{Y_{w}}\right)$—Weighted mean center of the research object;

$\tilde{x_{i}},\;\tilde{{\;y}_{i}}$—Coordinate deviations of the research object from the mean center;

$\sigma_{x},\;\;\sigma_{y}$—Standard deviations along the *x*-axis and *y*-axis directions, respectively.

## 4. Results and Analysis

### 4.1. Experimental Scheme

In this study, the classification scheme was developed with reference to the Current Land Use Classification standard (GB/T 21010-2017) [58], while considering the actual conditions of the Jiangsu coastal zone and the spatial resolution of the remote sensing imagery. The LULC types were categorized into five classes: water body, forest–grassland, construction land, cultivated land, and unused land (Table 2). This classification system was adopted to represent the major LULC types and their long-term changes in the study area while maintaining reliable class discrimination at the spatial resolution of Landsat imagery. Previous studies have indicated that both the quantity and representativeness of training samples influence the performance of machine learning based remote sensing classification [59]. Sample points were manually selected and labeled through joint visual interpretation of the corresponding Landsat imagery and high-resolution Google Earth imagery, following the principles of uniformity, dispersion, and representativeness. 

Previous studies have adopted an empirical training sample range of 10–30 *n* samples per class, where *n* denotes the number of image bands, as a reference for determining training sample size in remote sensing image classification [60]. In this study, this empirical range was used as a reference for training sample design, together with the spatial extent and representativeness of each LULC class. For each study year, approximately 900 samples were collected. Except for unused land, the other four LULC classes each contained approximately 200 samples. The number of unused land samples was relatively smaller because this class occupied a limited proportion of the study area. The samples were then randomly divided into training and test samples, with 70% used for classifier training and 30% reserved for accuracy assessment.

Subsequently, seven classification schemes were designed to compare the impact of multi-source feature variables on accuracy. Among them, the first six schemes served as comparison, while scheme 7 represented the feature-optimized approach. The feature variables and scheme design are detailed in Table 3.

### 4.2. Feature Selection Results

In this study, SHAP values were employed to quantify the feature importance, while the Pearson correlation coefficient was applied to assess linear relationships between features, and feature pairs with an absolute correlation coefficient greater than 0.8 were selected. Afterwards, for each highly correlated feature pair, the feature with lower SHAP importance was eliminated to complete the preliminary screening. Accordingly, the RFE method was further applied to iteratively refine the feature set and ultimately determine the optimal feature combination.

**Figure 3:**
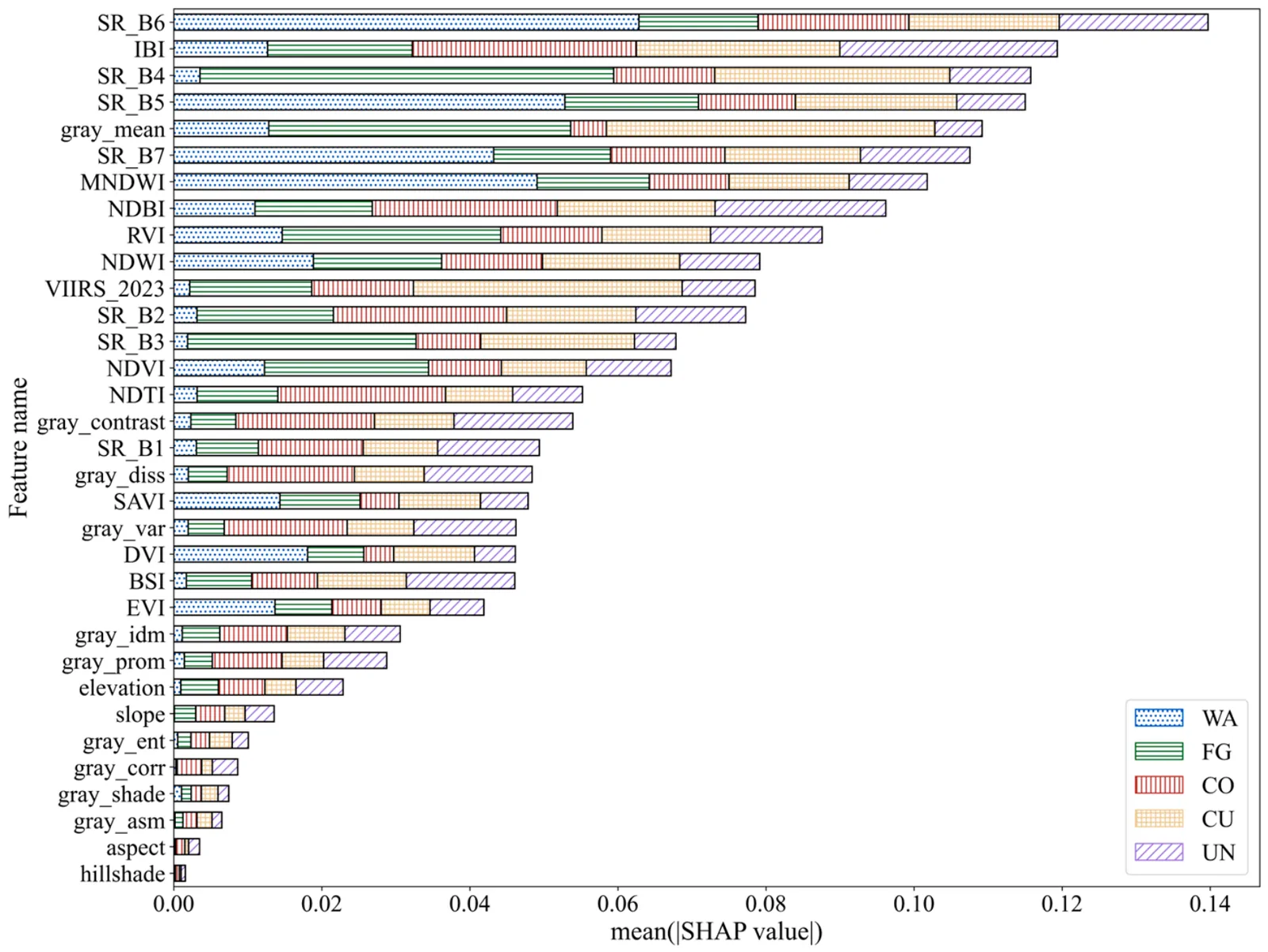
Feature importance ranking plot.

The SHAP interpretability framework evaluates the degree of influence of each feature on the model’s classification decision results by quantifying its average marginal contribution. A higher contribution value indicates greater feature importance. In this study, based on the 2023 data, the SHAP method was introduced to analyze feature importance in four models: RF, XGBoost, CART, and SVM. Figure 3 shows the average SHAP values of all features from the RF model. Bar length corresponds to contribution level, and features are ranked by significance. As shown in Figure 3, among the spectral features, SR_B6 demonstrated the highest importance for coastal LULC classification and is the most important feature determining the prediction results of the RF model. SR_B6 belongs to the shortwave infrared region and is highly sensitive to surface moisture. This property enhances the spectral contrast between water bodies and surrounding terrestrial surfaces. Xu [61] demonstrated that incorporating a shortwave infrared band into a water related spectral index can enhance open water features and reduce interference from built-up land, vegetation, and soil backgrounds. The complex LULC composition of the Jiangsu coastal zone likely further increased the discriminatory value of these spectral characteristics, contributing to the relatively high importance of SR_B6 in the classification. IBI ranked second in global feature importance, significantly contributing to the prediction of both construction land and unused land. Among texture features, gray_mean has a relatively high mean SHAP value, with a greater contribution rate to classification than other texture features, and shows a high contribution to cultivated land prediction. In contrast, aspect and hillshade ranked lowest in feature importance, which is mainly related to the generally flat terrain and limited topographic relief of the Jiangsu coastal zone. Aspect characterizes slope orientation, whereas hillshade represents variations in terrain illumination determined by topography and solar geometry. In low-relief plains, spatial variations in slope orientation and terrain-induced shading are relatively weak. Moreover, multiple LULC classes, including CU, CO, and FG, occur under similar flat topographic conditions. Consequently, aspect and hillshade provide limited inter-class discrimination and contribute relatively little additional information to the classification.

**Figure 4:**
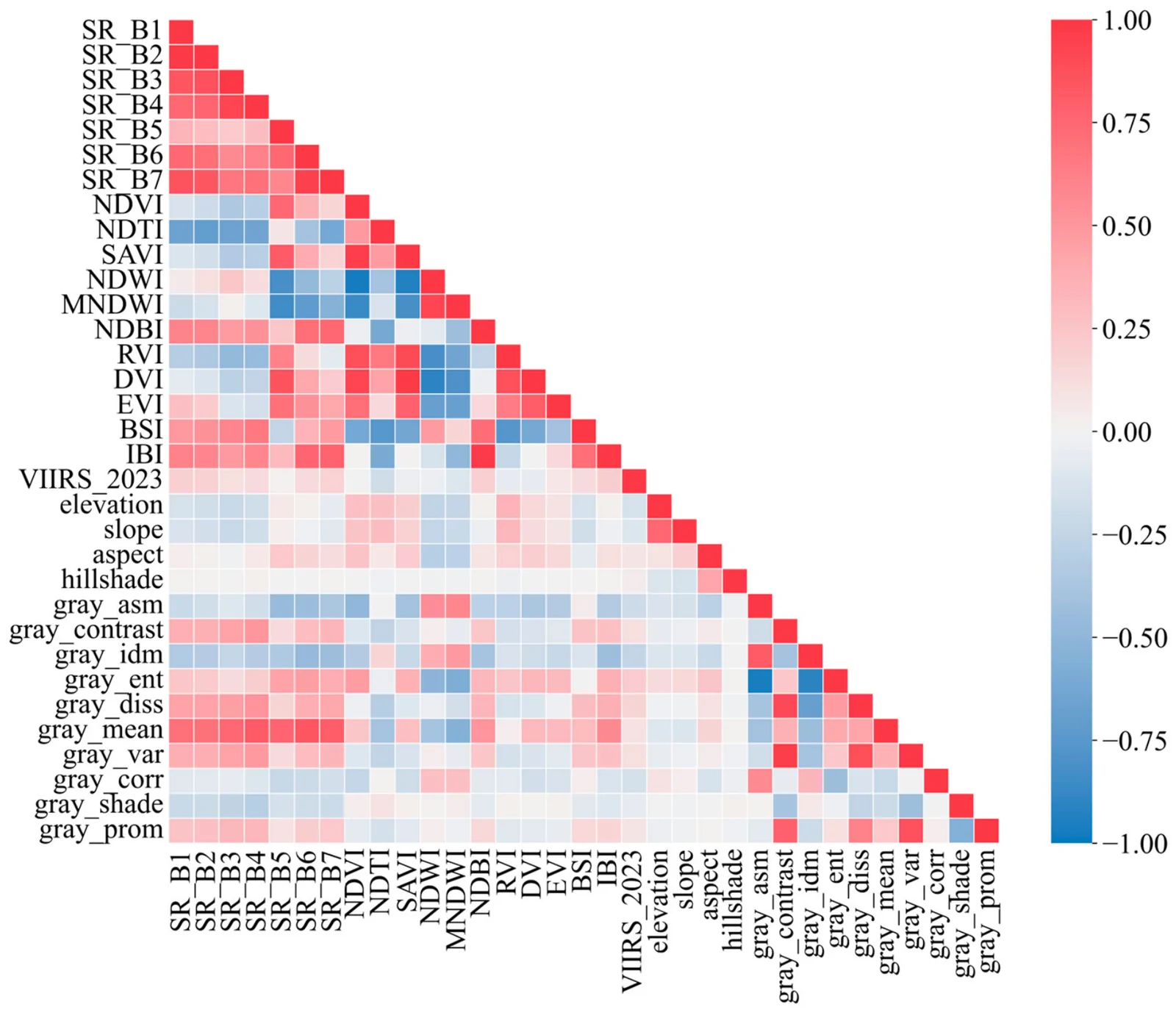
Correlation analysis of characteristic variables.

Overall, for LULC classification in coastal zone, the feature can be ranked by contribution in descending order as follows: spectral features, nighttime light features, texture features, and topographic features. Among them, spectral features such as SR_B6, IBI, SR_B4, and SR_B5 rank highly, indicating their significant contribution to classification. Crucially, SHAP analysis reveals that SR_B6 contributed most to WA identification, SR_B4 to FG identification, IBI to both CO and UN identification, and gray_mean to CU identification. These key variables with distinct physical significance were effectively retained in the final optimized feature subset determined by the Pearson-SHAP-RFE framework. Figure 4 illustrates the correlation matrix among the extracted characteristic variables.

### 4.3. Classification Results and Analysis

#### 4.3.1. Comparison of Classification Accuracy

The results of different feature combination schemes and classification algorithms are shown in Table 4. Scheme 1 employs only traditional spectral features, yielding the lowest accuracy, with OA values of 87.13%, 84.19%, 80.51%, and 86.40% for the RF, XGBoost, CART, and SVM models respectively. This suggests that relying exclusively on spectral information is inadequate to fully capture the intricate characteristics of LULC classes. The incorporation of texture, topographic, and nighttime light features generally im-proved the classification performance. For instance, in scheme 6, the OA values for the four algorithms reach 90.44%, 86.76%, 84.56%, and 91.18% respectively. This indicates that the fusion of multi-dimensional features can effectively capture the spatial and structural information of LULC classes, thereby improving the classification accuracy. However, it also increases the complexity and computational time of the model, reducing classification efficiency. Feature selection helps reduce redundant features and improve the model’s classification accuracy. Scheme 7 represents the feature-optimized scheme. After feature optimization, the numbers of features for the four algorithms were reduced from 33 to 15, 11, 8, and 11, respectively. The experimental results show that among the four classification algorithms, Scheme 7 consistently achieved the best classification performance, with OA values of 92.28%, 88.24%, 84.93%, and 91.54% for RF, XGBoost, CART, and SVM, respectively. This reveals that the feature selection method proposed in this study can reduce model complexity while maintaining high classification accuracy, thereby improving overall efficiency.

**Figure 5:**
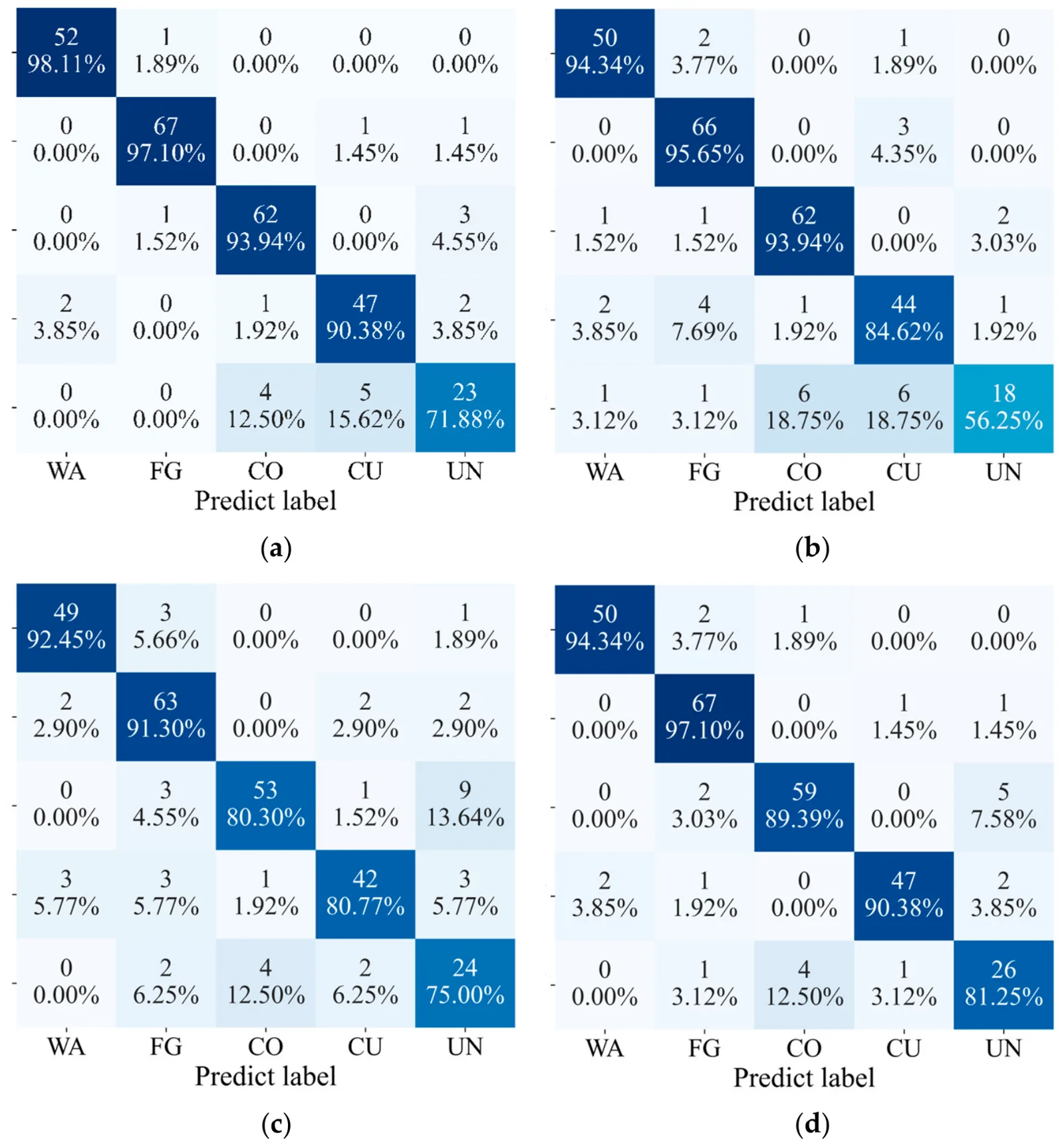
Confusion matrices of the four classification models: (**a**) RF; (**b**) XGBoost; (**c**) CART; (**d**) SVM.

Figure 5 illustrates the confusion matrices of the optimal models for the four classification algorithms. It is noted that the RF algorithm achieves the highest accuracy after feature selection. This model has fewer off-diagonal elements, indicating fewer misclassifications and better overall classification performance. This confirms that the RF model is both more accurate and reliable for coastal LULC classification compared to the other algorithms. Analysis of all four confusion matrices reveals a common misclassification pattern: unused land is frequently confused with construction land and cultivated land. Inaccuracy likely stems from the similar spectral characteristics and spatial intermingling of these LULC classes. In addition, some cultivated land is misclassified as unused land, which can be attributed to fallowing or variations in crop growth cycles that cause the spectral signals of cultivated plots to resemble those of bare soil.

#### 4.3.2. Analysis of Classification Results

**Figure 6:**
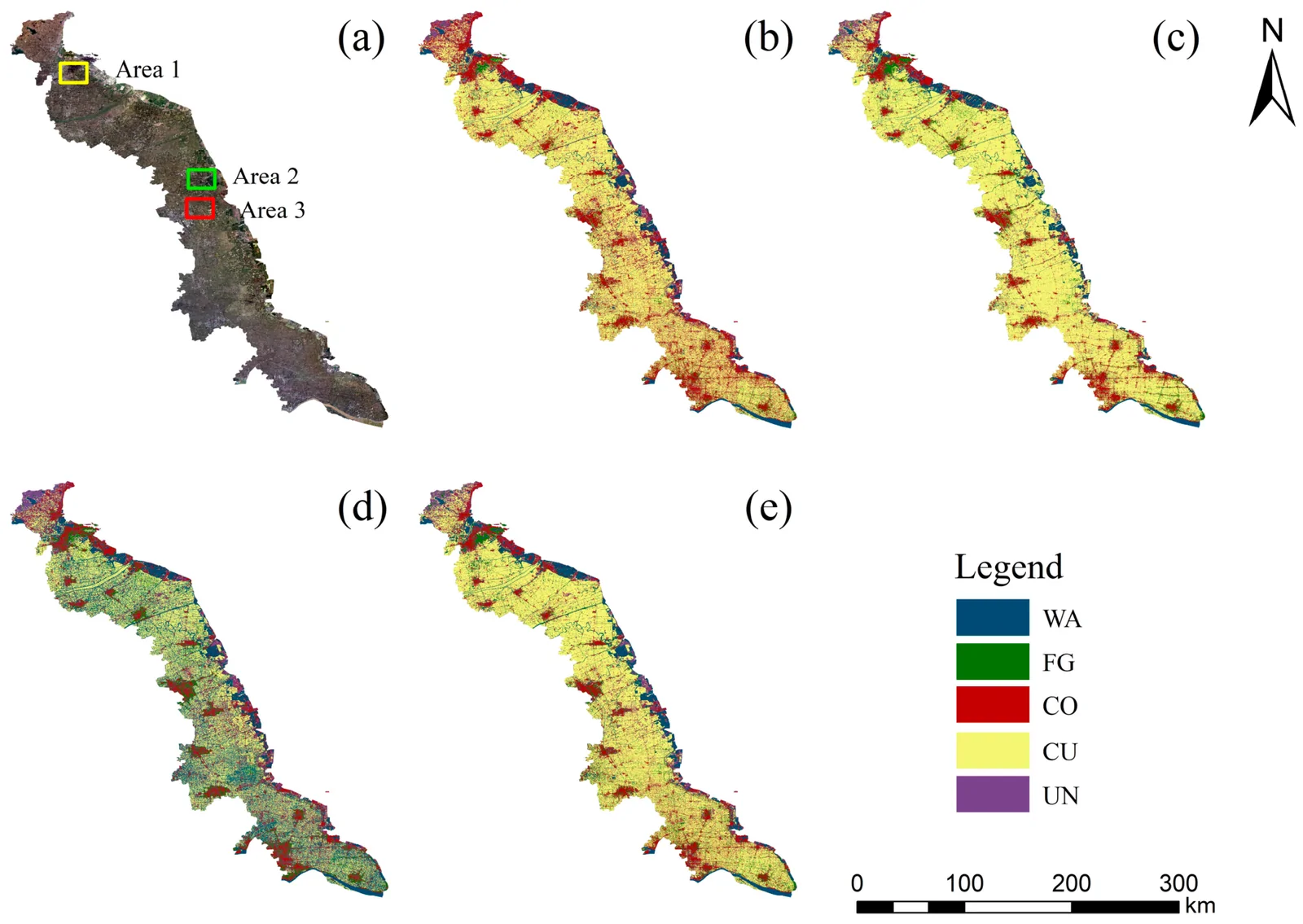
Classification results of different classification models: (**a**) Original image; (**b**) RF Classification; (**c**) XGBoost Classification; (**d**) CART Classification; (**e**) SVM Classification.

The study compared the highest-accuracy models from four classification algorithms following feature optimization. The corresponding accuracy assessment results and LULC classification maps of each model are exhibited in Figure 6 and Table 5. While the spatial distribution patterns across the four classification maps were basically consistent, the classification result obtained by the RF model showed superior clarity and spatial continuity of class patches. Meanwhile, the RF model achieved the highest classification accuracy after feature optimization, with an OA of 92.28% (95% CI: 88.97–95.59%), a Kappa coefficient of 0.9019 (95% CI: 0.8587–0.9431), and an F1-score of 0.9218 (95% CI: 0.8867–0.9548). SVM followed closely, with an OA of 91.54% (95% CI: 87.87–94.49%), a Kappa coefficient of 0.8927 (95% CI: 0.8467–0.9302), and an F1-score of 0.9158 (95% CI: 0.8807–0.9456). The classification accuracy of water bodies and forest–grassland exceeded 85% with water bodies achieving over 90% accuracy. In contrast, unused land had the lowest classification accuracy, likely due to its limited sample size, which hindered the model’s ability to learn its characteristics effectively. Overall, the RF model achieved the best classification performance, while CART showed more substantial misclassification, consistent with the confusion matrices presented in Figure 5.

To further assess the statistical significance of performance differences among the four classifiers, pairwise comparisons were conducted using OA as the comparison metric and paired bootstrap resampling, as summarized in Table 6. Statistically significant differences in OA were observed between RF and XGBoost (95% CI: [0.0074, 0.0735]), between RF and CART (95% CI: [0.0331, 0.1140]), and between SVM and CART (95% CI: [0.0221, 0.1103]). Conversely, the comparisons between RF and SVM, SVM and XGBoost, and XGBoost and CART did not show statistically significant differences, as their corresponding 95% confidence intervals included zero. In particular, although RF achieved a slightly higher OA than SVM, the difference between the two classifiers was not statistically significant.

**Figure 7:**
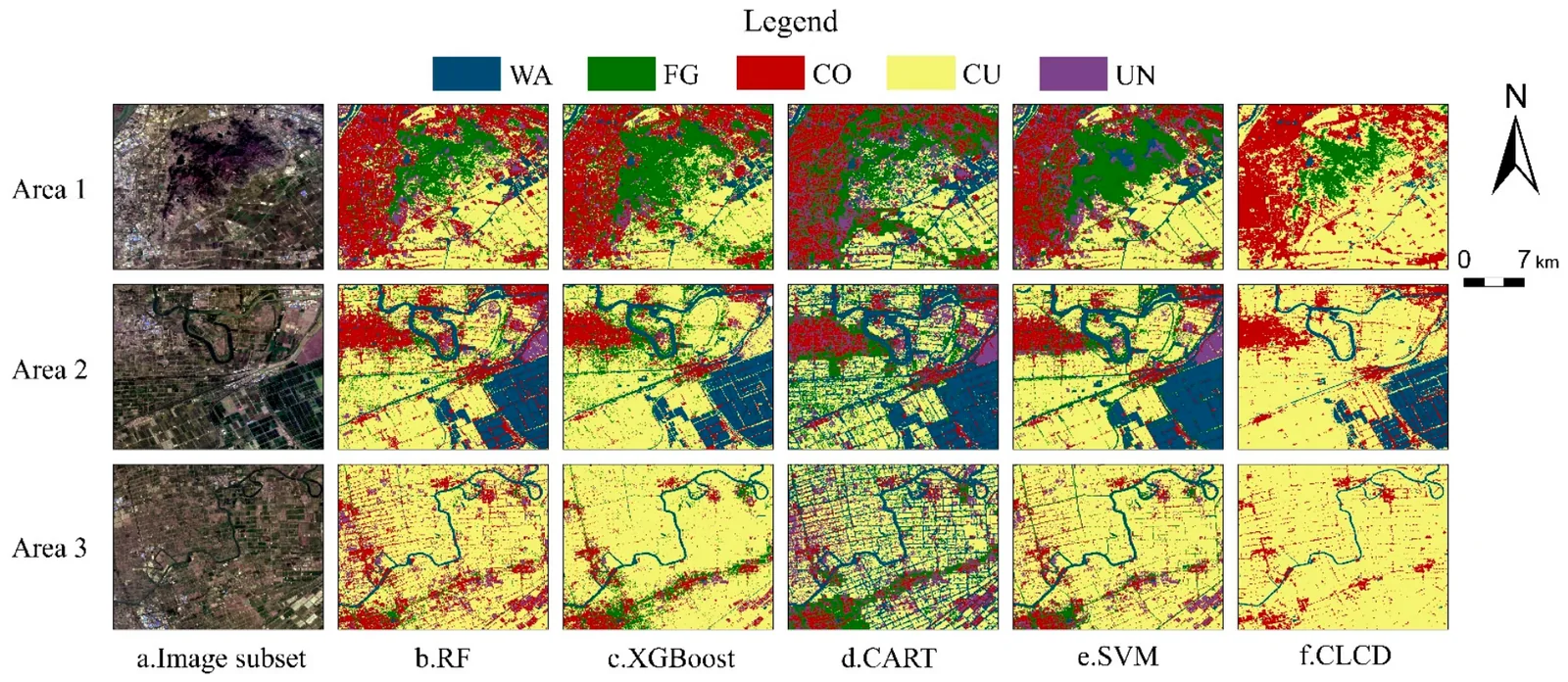
Partial map of Different classification models.

To further compare the classification performance of different schemes, this investigation extracted local enlargements of the classification results (Figure 7). The detailed views demonstrate that the RF model’s output aligns most closely with the actual LULC distribution. For example, in Area 1, the SVM result showed scattered water patches within the forest–grassland area, whereas the RF result preserved a more continuous forest–grassland pattern. In Area 2, the XGBoost model misclassified a section of unused land as cultivated land; the CART algorithm achieved the worst classification performance, erroneously classifying segments of construction and cultivated land as forest–grassland. Considering its overall classification performance, together with the lower degree of misclassification and better spatial continuity of the classification results, the feature-optimized RF model was selected for the subsequent long-term LULC classification.

The strong overall performance of RF may be related to its robustness in handling diverse remote sensing features and heterogeneous coastal landscapes. RF has been reported to effectively accommodate multicollinearity among predictor variables and to remain relatively insensitive to overfitting [36]. This property is relevant to the present study because spectral bands, spectral indices, texture, topographic, and nighttime light features were jointly incorporated, some of which are partially correlated. In addition, previous LULC studies have demonstrated that RF is robust to noise in heterogeneous landscapes [62]. Such robustness is particularly beneficial in the Jiangsu coastal zone, where fragmented LULC patterns and spectral overlap among classes complicate class discrimination. These characteristics likely contributed to the strong classification performance of RF observed in this study. 

To further verify the reliability of our classification, we conducted a cross-comparison between the 2023 RF results and the China Land Cover Dataset (CLCD). Based on the 30% independent validation samples, the consistency analysis yielded an OA of 68.54% and a Kappa coefficient of 0.5975. This moderate agreement indicates that while our results align with national-scale patterns, significant regional advantages exist. As demonstrated in the local enlargements (Figure 7), the CLCD product tends to exhibit over-smoothing or spectral confusion in complex coastal regions, failing to capture the spatial nuances identified by our proposed framework. Comparative analysis reveals that the RF model maintains a higher degree of spatial consistency with CLCD than the XGBoost and CART algorithms, while simultaneously providing superior fine-grained details in characterizing coastal landscapes.

#### 4.3.3. Analysis of Different Dimensionality Reduction Results

**Figure 8:**
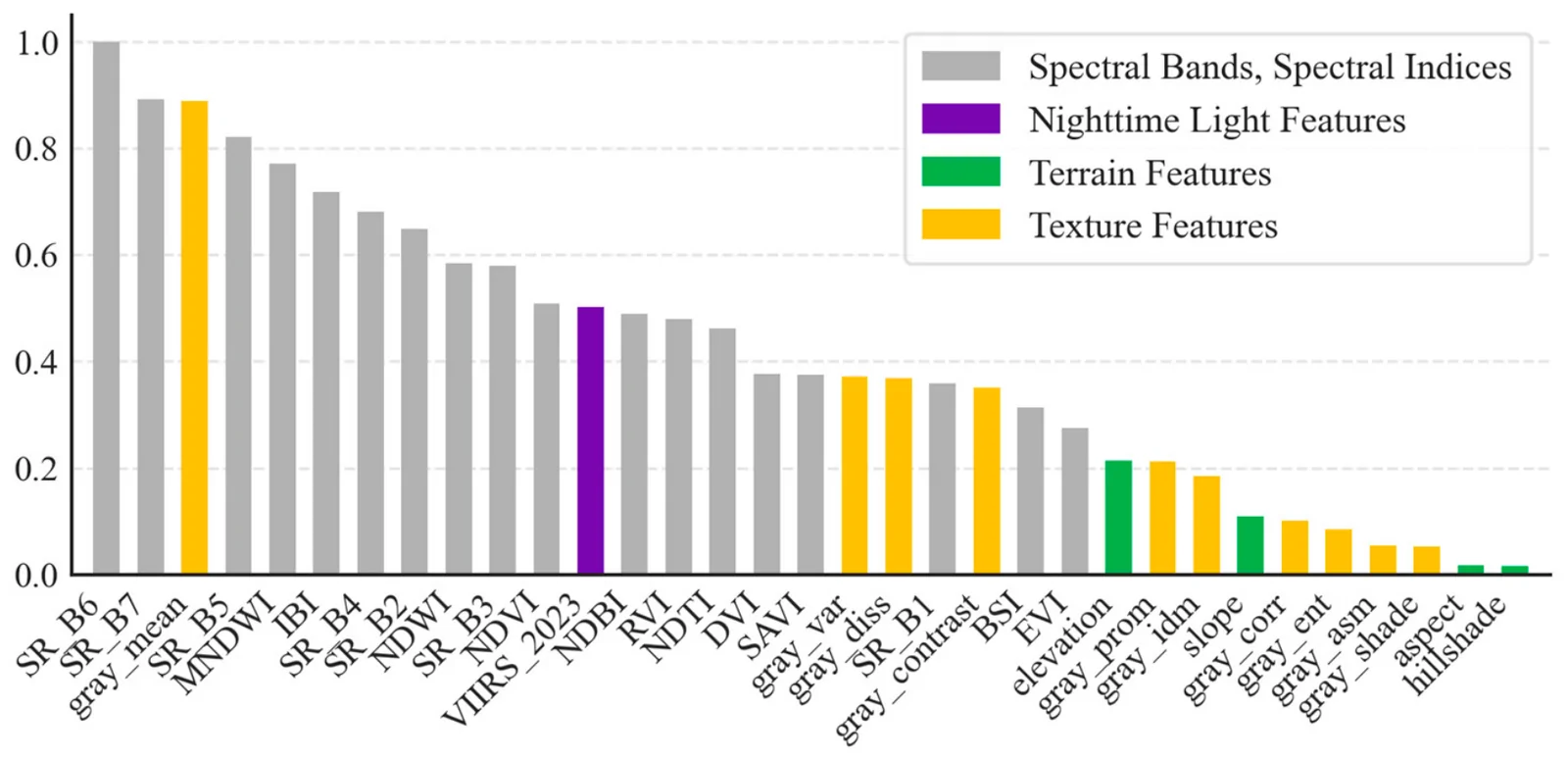
Feature importance evaluation of multi-source features based on Random Forest Gini index.

The Pearson-Gini-RFE utilized the Random Forest algorithm to calculate the importance of the initial 33 features, followed by a normalization process (as shown in Figure 8). After RFE screening, 11 optimal features were retained, achieving an overall accuracy of 90.81% and a Kappa coefficient of 0.8834. Comparing these results with the SHAP importance displayed in Figure 3, Gini importance and SHAP values maintained basic consistency in identifying dominant features, with spectral bands (e.g., SR_B6, SR_B7) predominating under both metrics. However, it is noteworthy that the Gini index only reflects the global contribution of features and lacks the granularity to detail feature performance across specific categories. In contrast, the SHAP method offers superior interpretability; it provides both a global importance perspective and accurately quantifies the local contribution of features to specific LULC class identification. For instance, SR_B6 contributed most to WA identification, while IBI showed the highest contribution to both CO and UN recognition. Under both importance metrics, spectral and Nighttime Light features with clear physical meanings ranked higher, texture features primarily played a supplementary role, and topographic features contributed relatively less due to the flat terrain of the study area. Overall, the ranking of the same feature remained fundamentally consistent across different importance assessment metrics, indicating that these features possess stable influence and predictive value.

**Figure 9:**
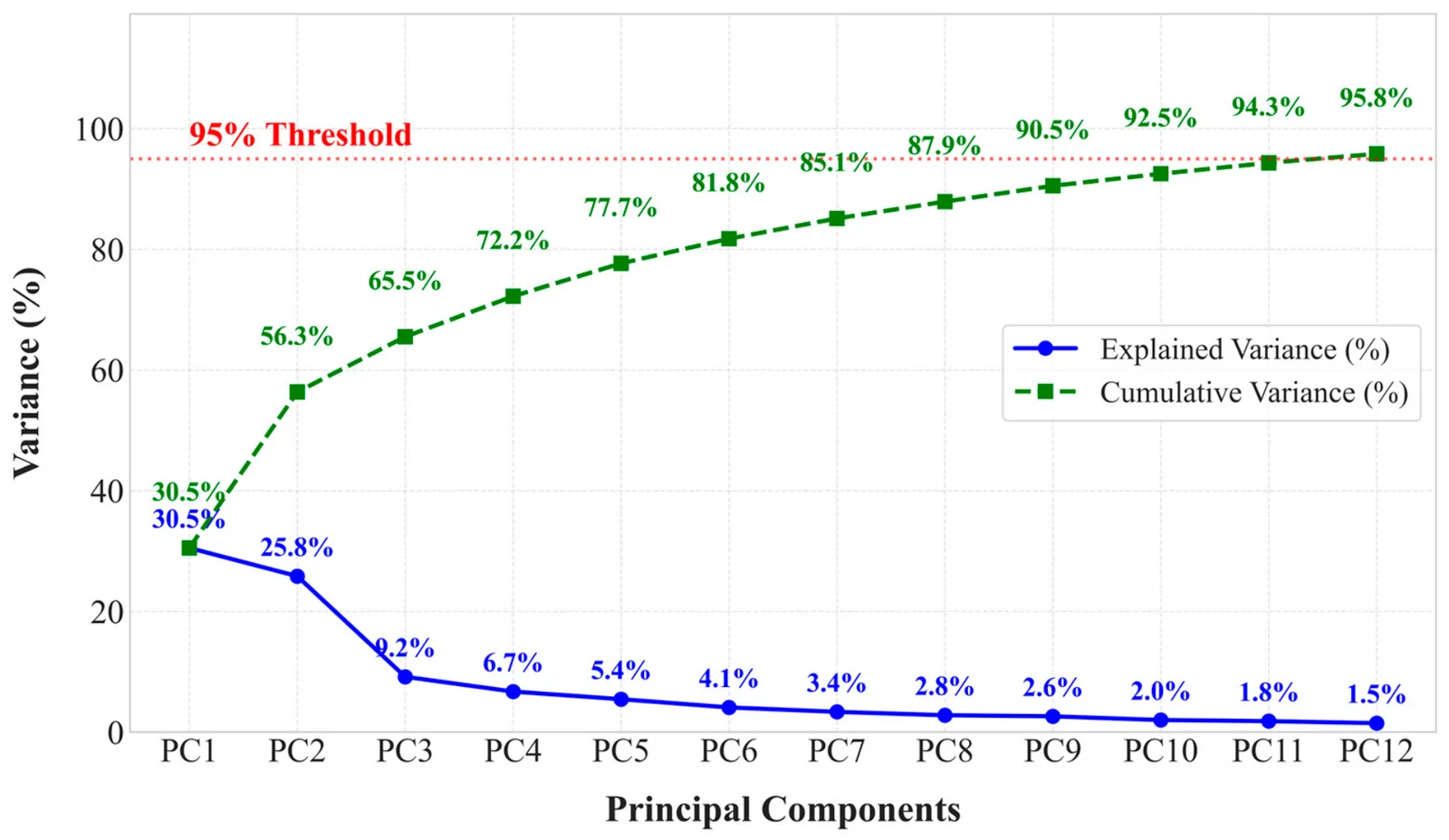
Individual and cumulative explained variance ratios of PCA.

As a widely adopted machine learning method, PCA is an established technique for data dimensionality reduction. In this study, Z-score normalization was first applied to the 33 constructed multi-source features to eliminate the interference of different scales on feature variance weights. Subsequently, a global PCA transformation was performed on the normalized matrix, projecting the original features into a linearly independent low-dimensional subspace through orthogonal transformation. According to the principle of cumulative variance contribution, 12 principal components were retained to construct a new feature set (as shown in Figure 9) to ensure 95% integrity of the original information. The analysis indicates that the variance contribution of the first principal component (PC1) is only 30.5%, far below the dominant levels typically observed in pure spectral data. This long-tail distribution characteristic reveals significant heterogeneity and complementarity among the multi-source data in the study area. Ultimately, the classification experiment based on PCA-reduced features achieved an OA of 91.54% and a Kappa coefficient of 0.8927. Although PCA effectively compresses feature dimensions, its generated principal components are linear combinations of the original variables, leading to the obscuration of physical meanings. Compared with the SHAP method, which provides intuitive visualization and local interpretability, PCA struggles to quantify the independent contribution of a single physical feature to the identification of specific LULC classes.

### 4.4. Spatiotemporal Changes in LULC in the Coastal Area

**Figure 10:**
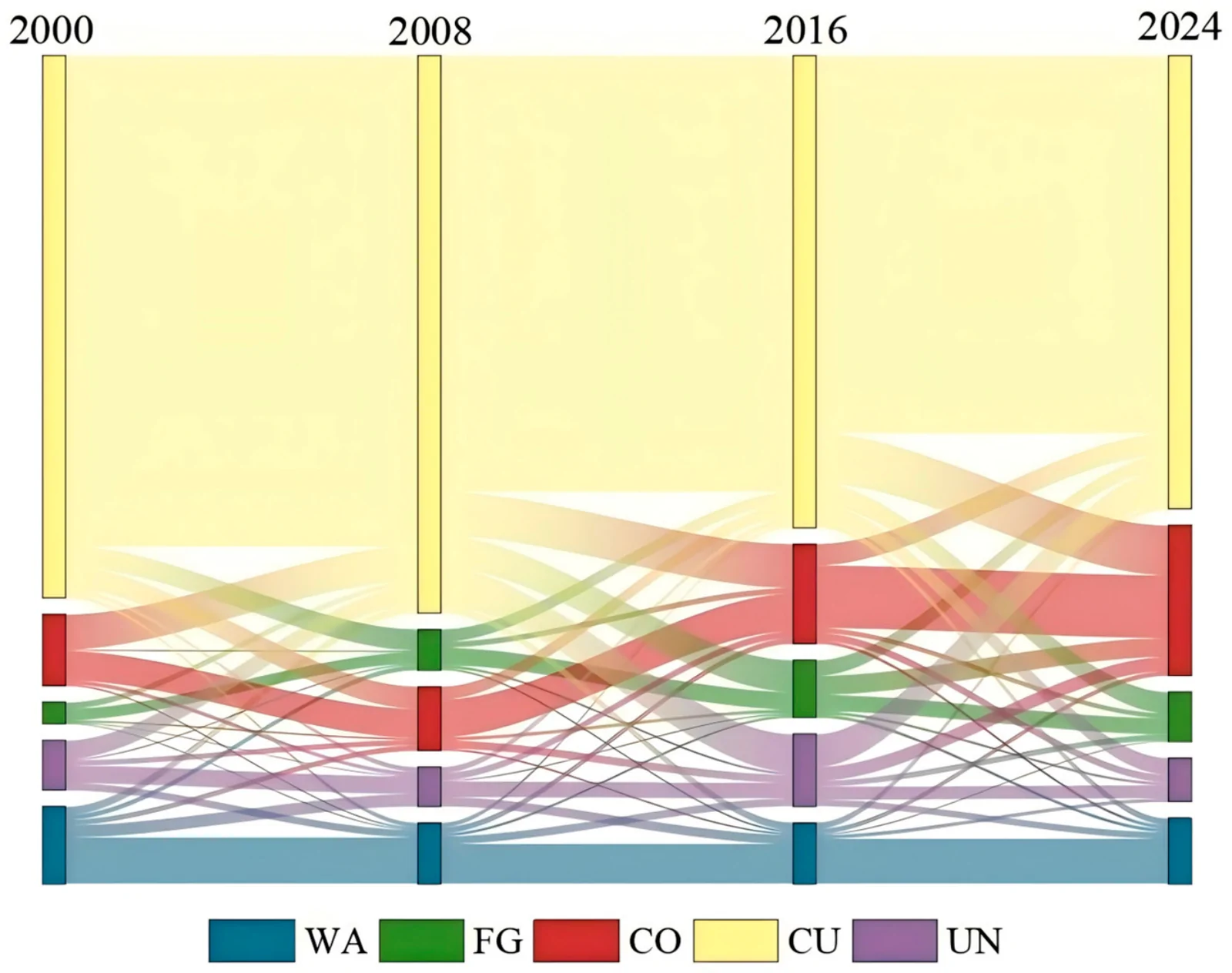
Sankey diagram of land use change in the coastal zone from 2000 to 2024.

Based on the 2023 accuracy evaluation, the multi-feature combination method with the highest accuracy was adopted, namely the one after feature optimization, which incorporated spectral bands, spectral indices, texture, topography, and nighttime light features. Using the RF algorithm, the land type distributions in the coastal zone in 2000, 2008, 2016, and 2024 were extracted respectively. Using the classification results, the land use transfer matrix was calculated, and the Sankey diagram of land use transitions was generated (Table 7 and Figure 10).

Analysis of land use transfer matrices for the three periods indicates that significant changes occurred primarily among forest–grassland, construction land, and cultivated land. The overarching trend was marked by a net decrease in water bodies, cultivated land and unused land, contrasted with a net increase in forest–grassland and construction land. Construction land exhibited an overall increasing trend over 2000–2024, with particularly pronounced expansion after 2008. Although cultivated land persisted as the predominant land use type overall, a consistent pattern of conversion to construction land and forest–grassland was observed, directly reflecting the competing regional demands of urban development and ecological construction. To intuitively visualize these complex land use fluxes, a Sankey diagram was generated based on the land use transfer matrix (Figure 10). This visualization effectively delineates the dominant conversion pathways between land use types, underscoring the central trends of construction land expansion, cultivated land reduction, and forest–grassland increase. These spatial and structural changes are closely associated with regional urbanization and the implementation of ecological conservation strategies.

**Figure 11:**
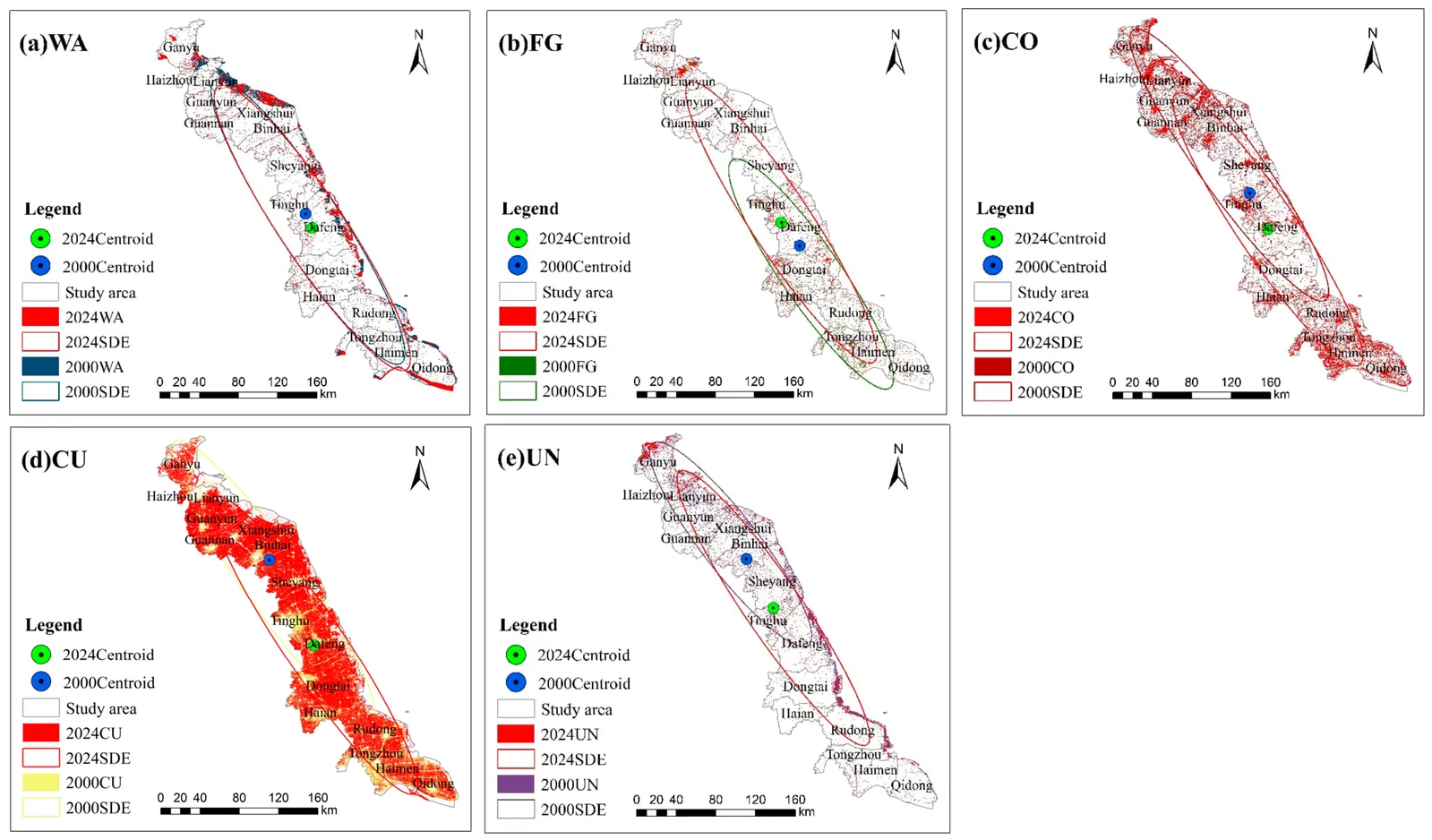
Standard deviational ellipses of the five LULC classes in the Jiangsu coastal zone from 2000 to 2024: (**a**) SDE of WA; (**b**) SDE of FG; (**c**) SDE of CO; (**d**) SDE of CU; (**e**) SDE of UN.

This study further employed standard deviation ellipse and centroid migration in ArcGIS 10.8 to quantify the spatial dynamics of LULC classes in the coastal zone (Table 8, Figure 11). Within this methodology, the major axis of the standard deviation ellipse indicates the spatial distribution direction of LULC classes in the Jiangsu coastal zone, where shorter length signifies a more concentrated distribution. Furthermore, a greater difference between the major and minor axes indicates stronger directional dominance in the spatial pattern.

From 2000 to 2024, the standard deviation ellipse for water bodies expanded slightly, with the major axis increasing by 0.53 km, and minor axis by 0.38 km, indicating high spatial stability. The minimal change in the axes suggests no pronounced directional shift in its distribution. The centroid of WA, consistently located in Dafeng District, migrated southward by 8.05 km. In contrast, the FG areas showed significant expansion, with the major axis lengthening 29.48 km. The difference between the lengths of the major and minor axes in 2000 changed significantly compared with that in 2024, indicating obvious transfer directionality. The centroid of FG remained consistently located in Dafeng District, migrating northward by 60.72 km during the study period. For CO, the length of the major axis of the standard deviation ellipse decreased by 1.95 km, with a slight change in the distribution direction; the length of the minor axis shortened by 0.42 km. The relatively stable axial difference implies a lack of strong directional bias. The centroid of construction land shifted from Sheyang County to Dafeng District, migrating southward by 75.74 km during the study period, which indicates substantial changes in its spatial distribution range. For cultivated land, the length of the major axis of the standard deviation ellipse increased by 8.58 km, while the length of the minor axis increased by 0.40 km. The centroid of cultivated land shifted from Binhai County to Dafeng District, migrating southward by 77.73 km during the study period. Cultivated land underwent the most dramatic transformation. Unused land also underwent a relatively significant transformation, with major axis expanding by 38.39 km and minor axis unchanged, shifting southward by 77.59 km from Binhai County to Sheyang County, representing a considerable spatial redistribution range.

## 5. Discussion

Among the four classifiers, RF achieved the highest values of OA, Kappa coefficient, and F1-score. Although the paired bootstrap analysis did not provide evidence of a statistically significant difference in OA between RF and SVM, statistically significant differences were observed between RF and both XGBoost and CART. In comparison, SVM showed a statistically significant difference only relative to CART, while its difference from XGBoost was not statistically significant. Considering these accuracy results together with the lower degree of misclassification and better spatial continuity observed in the classification maps, RF was therefore selected for the subsequent long-term LULC mapping. The classification accuracy obtained in this study also compared favorably with previous RF applications in coastal environments [63,64]. These results further verify the reliability and applicability of the RF model in coastal LULC classification. Despite this high performance, classification errors persisted, mainly involving confusion between cultivated land, construction land, and unused land. The highest misclassification rate was observed between unused land and cultivated land (15.62%), attributable to their spectral similarity during fallow periods or when unused land is temporarily cultivated. This misclassification phenomenon is consistent with the common problem identified by Mao et al. [65] in their research on land cover classification in Qianjiangyuan National Park. The misclassification rate between unused land and construction land was 12.50%, likely due to anthropogenic disturbance on bare soil, which alters its spectral signature to resemble impervious surfaces. However, the inherent limitations of the Pearson-SHAP-RFE framework in handling boundary samples warrant further discussion. Although the framework effectively minimizes redundancy, the exclusion of certain spectral indices with weak contributions during RFE may lead to the loss of subtle phenological information. For instance, the misclassification of CU and UN during fallow periods suggests that while the selected features capture the primary physical differences, they are less sensitive to the transient surface moisture fluctuations common in coastal reclaimed areas. This indicates that model optimization is a trade-off between global robustness and local sensitivity to class boundaries.

The moderate agreement between the RF classification results of this study and CLCD indicates that the two datasets are generally consistent in their overall spatial patterns, although local discrepancies remain. These differences may mainly arise from variations in classification frameworks and data processing strategies. CLCD is designed for annual land cover mapping at the national scale using a unified classification workflow, whereas this study focuses specifically on the Jiangsu coastal zone, where training samples were constructed according to regional LULC characteristics and classification was performed using an optimized set of multiple feature types. In addition, the two approaches differ in their use of temporal Landsat information and image compositing strategies, and consequently the construction of training samples and visual interpretation criteria are not entirely identical. Furthermore, the original CLCD classification scheme differs from the five-class system adopted in this study, and the required class reaggregation may also contribute to the observed discrepancies. In the local comparisons shown in Figure 7, the RF result also exhibited relatively higher spatial consistency with CLCD than the SVM, XGBoost, and CART results, which is consistent with its strong overall classification performance.

This study validates the advantages of the Pearson-SHAP-RFE framework for complex LULC classification through a comparative analysis of different dimensionality reduction strategies. While PCA effectively projects high-dimensional data onto maximum variance axes via coordinate transformation, it suffers from fundamental constraints: the directions of maximum variance are not necessarily optimal for classification, and the method fails to incorporate class label information [66]. In contrast, our Pearson-SHAP-RFE framework preserves the integrity of the original feature space, ensuring that the selected optimal subset remains physically meaningful. Unlike the Gini index, which provides only a generalized global ranking and often overlooks category-specific influences, the SHAP-based evaluation explicitly identifies the primary drivers for each LULC class. The SHAP-based evaluation provides critical insights into the physical mechanisms governing the identification of coastal ground objects, moving beyond simple ‘black-box’ rankings. Specifically, the high contribution of SR_B6 to WA identification is attributed to its superior sensitivity to water absorption, which effectively enhances the spectral contrast between water bodies and other terrestrial features, thereby mitigating common confusion in coastal spectroscopy. The high contribution of IBI to both CO and UN recognition suggests that this index is particularly informative for characterizing built-up and bare surface conditions in the coastal zone, where these LULC types often exhibit complex spectral responses. Furthermore, the high contribution of gray_mean to CU recognition suggests that average gray-level characteristics derived from texture analysis may provide complementary information for distinguishing cultivated land when spectral responses vary across crop growth stages.

In terms of temporal evolution, land use changes along the Jiangsu coast exhibited distinct stage-specific characteristics, closely associated with shifts in regional development strategies and ecological protection policies. During 2009–2016, the conversion of wetlands to CO was primarily driven by the ‘National Jiangsu Coastal Development Plan’ (2009). This era involved large-scale reclamation projects, such as the expansion of Lianyungang and Dafeng Ports, which reshaped the coastline and altered local land-use patterns—a trend consistent with Li et al. [67] (2022). In contrast, the post-2016 period marks a shift toward ecological restoration. Projects like the ‘Spartina alterniflora management’ and the revised Jiangsu Land Regulation (revised in 2021) introduced strict ‘ecological red lines,’ facilitating the systematic recovery of FG from reclaimed land. Overall, the temporal evolution of land use during the study period reflects a transition along the Jiangsu coast from relatively intensive development toward ecological protection and restoration, while these stage-specific temporal changes also provide an important context for understanding the spatial centroid migration of different LULC types.

In terms of spatial evolution, the standard deviation ellipse analysis showed that the centroid of water bodies migrated only 8.05 km southward, whereas the centroid of cultivated land shifted southward from Binhai County to Dafeng District, with a migration distance of 77.73 km. The relatively small centroid displacement of water bodies indicates that their overall spatial distribution remained relatively stable. During the study period, Jiangsu continuously strengthened the protection of water areas. The Measures of Jiangsu Province for the Administration of Water Area Occupation by Construction Projects (2013) explicitly required preventing the reduction in existing water areas, maintaining the basic water-surface ratio, and implementing equivalent replacement for water areas occupied by construction projects. Subsequent water-area protection regulations were further strengthened, which, to some extent, helped maintain the spatial stability of rivers, lakes, and other water bodies. In contrast, the pronounced southward migration of cultivated land was mainly associated with the expansion of construction land and the development of coastal tidal flats. Some existing cultivated land was converted to construction land during the expansion of built-up areas. Meanwhile, the Opinions of the Jiangsu Provincial Government on Further Promoting the Scientific Development of Coastal Areas (2013) stipulated that newly reclaimed land suitable and intended for cultivation should be incorporated into supplementary cultivated land project management under the cultivated-land occupation–compensation balance policy, making coastal reclamation an important pathway for cultivated-land supplementation. Liang et al. [68] (2023) reported that parts of the reclaimed land in the Tiaozini Wetland, Dongtai, were used mainly for agriculture and aquaculture, and that the area of cultivated land increased by 14.03 km^2^ between 2014 and 2018, further indicating that agricultural development in the central and southern coastal reclamation areas played an important role in reshaping the spatial pattern of cultivated land. Together, these changes reflect the north and south redistribution of cultivated land within the study area.

## 6. Conclusions

This study established a robust methodological framework for monitoring LULC dynamics in the Jiangsu coastal zone utilizing the GEE platform and multi-source data from 2000 to 2024. By synergistically integrating Pearson correlation, SHAP importance, and Recursive Feature Elimination (RFE), we proposed a novel feature selection method and benchmarked it against traditional dimensionality reduction strategies to identify the optimal high-precision classification model. The main conclusions are as follows:

1. The integration of multi-source features—specifically nighttime light, texture, and topography—effectively compensates for spectral limitations, significantly enhancing the model’s ability to capture the spatial and structural nuances of complex coastal land–sea interaction zones.
2. The proposed Pearson-SHAP-RFE framework achieved favorable classification performance while preserving physical interpretability. Compared with Pearson-Gini-RFE and PCA, the proposed method retained the physical meaning of the original features while enabling both global feature importance assessment and class-specific interpretation, thereby providing further insight into the roles of key features in different LULC classes.
3. Over the past 24 years, the region’s LULC evolution reflects a profound transition from intensive anthropogenic exploitation to a policy-driven “ecological civilization”. The continuous decline in cultivated land due to urbanization contrasts with a phased recovery of forest–grassland driven by ecological restoration projects, illustrating the dynamic spatial interplay between regional economic expansion and environmental governance.

Although the Landsat surface reflectance products used in this study have undergone official atmospheric correction, local complex aerosol conditions in coastal land–sea interaction zones may still introduce certain uncertainties in surface reflectance, thereby affecting the spectral feature extraction of some coastal LULC types. In addition, as only four years were selected for analysis, some impacts of short-term rapid changes on the coastal zone may not be fully captured. Although annual median compositing reduces dependence on the acquisition date of individual images, it cannot completely eliminate seasonal spectral differences caused by vegetation phenology and crop growth cycles. Future studies could incorporate annual or even seasonal observations to better characterize short-term LULC dynamics. Although the training samples were interpreted by integrating 30 m Landsat imagery with high-resolution Google Earth basemaps to improve labeling accuracy as much as possible, some labeling uncertainties remain unavoidable due to visual interpretation limitations, unclear boundaries of certain LULC types, and spectral similarity among classes. Furthermore, this study adopted a five-class LULC classification scheme for regional-scale mapping. Limited by the 30 m medium spatial resolution of Landsat imagery and the availability of training samples, the capability to finely distinguish small-scale or spatially fragmented coastal features, such as salt marshes, Spartina alterniflora, and aquaculture ponds, remains constrained. Future studies could further improve coastal LULC mapping by integrating field survey data and high-resolution UAV imagery for classification validation and refinement, as well as incorporating super-resolution reconstruction techniques and multi-source heterogeneous data to enhance feature representation for complex coastal LULC identification. In addition, neural networks could be incorporated in future studies to further evaluate the generalizability of the proposed framework.

## Figures and Tables

**Table 1:** Calculation formulas for spectral indices.

Spectral Index	Formula	References
NDVI	(NIR − RED)/(NIR + RED)	[25]
NDTI	(SWIR1 − SWIR2)/(SWIR1 + SWIR2)	[26]
SAVI	((NIR − RED)/(NIR + RED + 0.5)) × 1.5	[27]
NDWI	(GREEN − NIR)/(GREEN + NIR)	[28]
MNDWI	(GREEN − SWIR1)/(GREEN + SWIR1)	[29]
NDBI	(SWIR1 − NIR)/(SWIR1 + NIR)	[30]
RVI	NIR/RED	[31]
DVI	NIR − RED	[32]
EVI	2.5 × (NIR − RED)/(NIR + 6 × RED − 7.5 × BLUE + 1)	[33]
BSI	((RED + SWIR1) − (NIR + BLUE))/((RED + SWIR1) + (NIR + BLUE))	[34]
IBI	[2 × SWIR1/(SWIR1 + NIR) − (NIR/(NIR + RED) + GREEN/(GREEN + SWIR1))]/[2 × SWIR1/(SWIR1 + NIR) + (NIR/(NIR + RED) + GREEN/(GREEN + SWIR1))]	[35]

**Table 2:** LULC classification system.

Category	Abbreviation	Description
Water body	WA	This refers to natural water bodies or artificially constructed water conservancy facilities; the study area primarily consists of rivers and reservoirs.
Forest–Grassland	FG	This refers to the land where trees, bamboo, shrubs grow, as well as the land along the coast where mangroves grow, and land mainly growing herbaceous plants.
Construction land	CO	This includes urban areas, rural areas, and industrial and mining land greatly affected by human activities.
Cultivated land	CU	This refers to the land used for farming and growing crops. The study area includes plain dry land, dry land and paddy fields.
Unused land	UN	This refers to land that has not been developed or is difficult to utilize. The study area is mostly bare land.

**Table 3:** Feature combination schemes.

Scheme	Feature Variable Combinations
1	Spectral bands + Spectral indices
2	Spectral bands + Spectral indices + Texture features
3	Spectral bands + Spectral indices + Terrain features
4	Spectral bands + Spectral indices + Nighttime light features
5	Spectral bands + Spectral indices + Texture features + Terrain features
6	All features
7	Optimized features

**Table 4:** Accuracy comparison of seven scheme classification algorithms.

Model	Accuracy	Scheme Name						
		1	2	3	4	5	6	7
RF	OA (%)	87.13	88.24	89.34	90.07	90.07	90.44	92.28
	Kappa	0.837	0.8504	0.865	0.8741	0.874	0.8784	0.9019
	F1-score	0.8715	0.8806	0.8937	0.9003	0.9005	0.9028	0.9218
XGBoost	OA (%)	84.19	87.50	83.83	85.66	87.13	86.76	88.24
	Kappa	0.7986	0.8409	0.7939	0.8172	0.8362	0.8311	0.8499
	F1-score	0.8367	0.8724	0.8329	0.8491	0.8688	0.8604	0.8778
CART	OA (%)	80.51	83.46	80.51	80.88	84.56	84.56	84.93
	Kappa	0.7538	0.7893	0.7538	0.7595	0.8045	0.8049	0.8094
	F1-score	0.8102	0.8351	0.8102	0.8174	0.8483	0.8475	0.8512
SVM	OA (%)	86.40	87.13	86.03	87.87	90.07	91.18	91.54
	Kappa	0.8285	0.8374	0.8234	0.8471	0.8742	0.8881	0.8927
	F1-score	0.8662	0.8735	0.8622	0.8815	0.9011	0.9116	0.9158

**Table 5:** Experimental accuracy of different classification models. Values in brackets represent the 95% bootstrap confidence intervals.

Category	RF		XGBoost		CART		SVM	
	UA (%)	PA (%)	UA (%)	PA (%)	UA (%)	PA (%)	UA (%)	PA (%)
WA	96.30	98.11	92.59	94.34	90.74	92.45	96.15	94.34
FG	97.10	97.10	89.19	95.65	85.14	91.30	91.78	97.10
CO	92.54	93.94	89.86	93.94	91.38	80.30	92.19	89.39
CU	88.68	90.38	81.48	84.62	89.36	80.77	95.92	90.38
UN	79.31	71.88	85.71	56.25	61.54	75.00	76.47	81.25
OA/%	92.28 [88.97–95.59]		88.24 [84.55–91.91]		84.93 [80.51–88.60]		91.54 [87.87–94.49]	
Kappa	0.9019 [0.8587–0.9431]		0.8499 [0.8011–0.8949]		0.8094 [0.7534–0.8562]		0.8927 [0.8467–0.9302]	
F1-score	0.9218 [0.8867–0.9548]		0.8778 [0.8349–0.9149]		0.8512 [0.8077–0.8889]		0.9158 [0.8807–0.9456]	

**Table 6:** Pairwise comparisons of overall accuracy based on paired bootstrap resampling.

Comparison	ΔOA	95% CI	*p*	Significance
RF vs. SVM	+0.0074	[−0.0184, +0.0331]	0.700	No
RF vs. XGBoost	+0.0404	[+0.0074, +0.0735]	0.016	Yes
RF vs. CART	+0.0735	[+0.0331, +0.1140]	<0.001	Yes
SVM vs. XGBoost	+0.0331	[+0.0000, +0.0662]	0.057	No
SVM vs. CART	+0.0662	[+0.0221, +0.1103]	0.002	Yes
XGBoost vs. CART	+0.0331	[−0.0147, +0.0809]	0.197	No

**Table 7:** Land use transfer matrix of the coastal zone from 2000 to 2024.

Period	Category/Area (km^2^)	WA	FG	CO	CU	UN
2000–2008	WA	1496.89	157.43	172.78	260.60	398.68
	FG	35.64	367.67	68.50	197.40	23.17
	CO	60.55	61.09	937.84	1125.88	126.72
	CU	141.78	680.04	716.60	15,841.30	153.59
	UN	235.24	53.54	172.15	587.07	562.46
2008–2016	WA	1282.98	100.70	188.70	143.46	254.26
	FG	73.74	563.78	186.88	401.50	93.87
	CO	75.55	115.15	1212.81	411.78	252.59
	CU	244.97	1023.11	1419.20	14,105.59	1219.38
	UN	299.07	57.04	216.33	190.61	501.57
2016–2024	WA	1451.67	44.71	156.72	200.11	123.08
	FG	116.98	522.43	613.83	502.82	103.72
	CO	115.96	156.37	2027.12	690.60	233.87
	CU	211.52	689.20	1604.76	12,208.51	538.94
	UN	213.55	188.07	448.42	1053.29	418.35

**Table 8:** Coastal zone center of gravity migration from 2000 to 2024.

Category	Gravity Center Coordinate (km)		Major Axis (km)		Minor Axis (km)		Rotation (°)		Migration Distance (km)
	2000	2024	2000	2024	2000	2024	2000	2024	
WA	814.78, 3683.19	819.28, 3676.51	172.96	173.49	32.61	32.99	146.64	146.32	8.05
FG	841.69, 3629.36	811.31, 3681.93	141.49	170.97	29.98	30.63	145.28	146.52	60.72
CO	782.25, 3739.98	821.34, 3675.11	168.14	166.19	30.47	30.05	144.41	145.92	75.74
CU	778.81, 3741.23	820.39, 3675.56	174.11	182.69	30.54	30.94	144.87	145.93	77.73
UN	758.71, 3777.69	801.85, 3713.20	128.85	167.24	28.41	28.41	142.28	145.59	77.59

## Data Availability

The original remote sensing datasets used in this study are publicly available and were accessed through the Google Earth Engine platform. These datasets include Landsat surface reflectance imagery, SRTM DEM data, and nighttime light products, including DMSP-OLS and NPP-VIIRS. The processed data and classification results generated in this study are available from the corresponding author upon reasonable request. No additional permission is required for the use of these publicly available datasets.
